# Anti‐β7 integrin treatment impedes the recruitment on non‐classical monocytes to the gut and delays macrophage‐mediated intestinal wound healing

**DOI:** 10.1002/ctm2.1233

**Published:** 2023-04-08

**Authors:** Katrin Sommer, Karin Heidbreder, Lucas Kreiss, Mark Dedden, Eva‐Maria Paap, Maximilian Wiendl, Emily Becker, Raja Atreya, Tanja M. Müller, Imke Atreya, Maximilian Waldner, Sebastian Schürmann, Oliver Friedrich, Markus F. Neurath, Sebastian Zundler

**Affiliations:** ^1^ Department of Medicine University Hospital Erlangen and Friedrich‐Alexander‐Universität Erlangen‐Nürnberg Erlangen Germany; ^2^ Institute of Medical Biotechnology Department of Chemical and Biological Engineering (CBI) Friedrich‐Alexander‐Universität Erlangen‐Nürnberg Erlangen Germany; ^3^ Deutsches Zentrum Immuntherapie (DZI) University Hospital Erlangen Erlangen Germany

**Keywords:** etrolizumab, gut homing, intestinal wound healing, monocytes, vedolizumab

## Abstract

**Background:**

Closing mucosal defects to reach mucosal healing is an important goal of therapy in inflammatory bowel disease (IBD). Among other cells, monocyte‐derived macrophages are centrally involved in such intestinal wound healing. We had previously demonstrated that the anti‐α4β7 integrin antibody vedolizumab blocks the recruitment of non‐classical monocytes as biased progenitors of wound healing macrophages to the gut and delays wound healing. However, although important for the interpretation of disappointing results in recent phase III trials in IBD, the effects of the anti‐β7 antibody etrolizumab on wound healing are unclear so far.

**Methods:**

We analyzed the expression of etrolizumab targets on human and mouse monocyte subsets by flow cytometry and assessed their function in adhesion and homing assays. We explored wound‐associated monocyte recruitment dynamics with multi‐photon microscopy and compared the effects of etrolizumab and vedolizumab surrogate (‐s) antibodies on experimental wound healing and wound‐associated macrophage abundance. Finally, we investigated wound healing macrophage signatures in the large intestinal transcriptome of patients with Crohn's disease treated with etrolizumab.

**Results:**

Human and mouse non‐classical monocytes expressed more αEβ7 integrin than classical monocytes and were a target of etrolizumab‐s, which blocked non‐classical monocyte adhesion to MAdCAM‐1 and E‐Cadherin as well as gut homing in vivo. Intestinal wound healing was delayed on treatment with etrolizumab‐s along with a reduction of peri‐lesional wound healing macrophages. Wound healing macrophage signatures in the colon of patients with Crohn's disease were substantially down‐regulated on treatment with etrolizumab, but not with placebo.

**Conclusions:**

Combined blockade of αEβ7 and α4β7 with etrolizumab seems to exceed the effect of anti‐α4β7 treatment on intestinal wound healing, which might help to inform further investigations to understand the recent observations in the etrolizumab phase III trial program.

## Graphical abstract

- A subset of non‐classical monocytes expresses αEβ7 integrin and is a target of etrolizumab‐s.
- Etrolizumab‐s blocks adhesion and gut homing of non‐classical monocytes.
- Etrolizumab‐s delays intestinal wound healing and reduces the presence of peri‐lesional macrophages.
- Wound healing macrophage signatures are down‐regulated upon etrolizumab treatment in patients with CD.

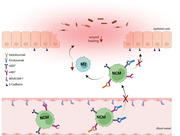

## 1 BACKGROUND

Inflammatory bowel diseases (IBD) comprise the main entities Crohn's disease (CD) and ulcerative colitis (UC).^1^, ^2^ They are chronic inflammatory conditions of the intestine that are considered to result from a multifactorial interplay of environmental factors with host susceptibility at the interface of a weakened gut epithelial barrier. Together, these pathogenetic events converge into aberrant activation of the intestinal immune system resulting in pro‐inflammatory signaling and tissue destruction.^3^

Medical interventions to treat IBD mainly target the immune cell compartment by blocking pro‐inflammatory cytokines, promoting apoptosis or inhibiting the recruitment of immune cells. If successful, this leads to restoration of homeostasis and mucosal healing.^4^

Such closure of mucosal defects relies on various tightly regulated and overlapping steps. During an early inflammatory phase phagocytes, such as neutrophils and macrophages are recruited to the intestinal wound area, where they oppose microbiota threatening to cross the barrier and neutrophils provisionally seal the defect by forming neutrophil extracellular traps.^5^ This phase gradually transitions towards a proliferative phase marked by restorative processes including neoangiogenesis and de novo‐formation of tissue. Finally, it is accomplished by a remodeling phase, in which the tissue matures and re‐achieves homeostasis.^6^

Given this complexity, it is not surprising that intestinal wound healing requires a balanced interplay of multiple cellular actors.^7^ One population that is of main interest in this context are macrophages, which appear in different activation states^8^ and fulfill different functions during these stages.^9^, ^10^ While pro‐inflammatory ‘M1‐like’ macrophages expressing molecules, such as iNOS, are crucial for the early phase of wound healing, ‘M2‐like’ macrophages with a wound healing phenotype expressing molecules such as Arginase or CD206 are involved in proliferation and remodeling.^9^, ^11^, ^12^ Although a population of locally self‐maintaining macrophages has recently been described in the gut,^13^ recruitment of monocytes, which locally differentiate into these macrophage fates, plays an important role for replenishing intestinal macrophage pools.^14^, ^15^ In humans, three different monocyte subsets are distinguished based on their surface expression of CD16 and CD14, while in mice classical monocytes (CLMs) and non‐classical monocytes (NCM) are divided based on their level of Ly6C and/or Cx3cr1 expression.^16^

Importantly, immune cell recruitment to the intestine is a target of drugs designed for the therapy of IBD.^17^ One of them is the anti‐β7 integrin antibody etrolizumab.^18^ It is considered to interfere with α4β7 integrin‐dependent gut homing of T cells and to additionally block T cell retention in the inflamed mucosa via disruption of the interaction of αEβ7 with epithelial E‐Cadherin.^19^, ^20^ While very promising results had been reported in a phase II trial in UC,^21^ only few primary endpoints were met in a large phase III trial program in UC.^22^, ^23^, ^24^, ^25^, ^26^

We had previously shown that the anti‐α4β7 antibody vedolizumab blocks the gut homing of non‐classical monocytes, which are skewed for differentiation into wound healing macrophages. This was associated with delayed intestinal wound healing.^27^

Here, we hypothesized that αEβ7 integrin‐dependent retention might also play a role for monocyte and macrophage trafficking in the gut and investigated the impact of anti‐β7 compared with anti‐α4β7 blockade on wound healing. We show that etrolizumab surrogate (etrolizumab‐s) more effectively decreases the accumulation of non‐classical monocytes in the gut, which is associated with delayed experimental wound healing and decreased presence of macrophages with a wound healing phenotype. RNA‐seq analysis suggested that similar mechanisms apply in humans.

Thus, our data indicate that the blockade of reparative processes in the gut by etrolizumab might be one piece of the puzzle to understand the observations made in clinical trials.

## 2 METHODS

### 2.1 Isolation of human peripheral blood cells

Peripheral blood samples from patients with UC and CD as well as healthy control donors were obtained at the Department of Medicine 1 of the University Hospital Erlangen after informed written consent according to the approval of the Ethics Committee of the Friedrich‐Alexander‐University Erlangen‐Nuremberg (426_20B). Patient characteristics are summarized in Table 1. Peripheral blood mononuclear cells (PBMCs) were isolated by density gradient centrifugation using LSM Lymphocyte Separation Medium (Anprotec).

**TABLE 1:** Patient characteristics

		Non‐IBD	CD	UC
*N*		15	14	12
**Age (mean, range)**		26.8 (19–41)	37.43 (23–64)	38.25 (22–66)
**Female (%)**		80	50	25
**Disease activity**	HBI (mean, range)		4.54 (1–12)	
	PMS (mean, range)			2.83 (0–7)
	CRP (mg/L) (mean, range)		18.75 (1.3–110.3)	4.54 (0.5–27.4)
**Therapy (%)**	Mesalazin		0	0
	Steroids		0	0
	Immunosuppresants		0	8.33
	anti‐tumor necrosis factor (TNF)		78.57	75
	Vedolizumab		7.14	8.33
	Ustekinumab		14.29	8.33
**Localisation (%)**			L1: 28.57	E1: 16.67
			L2: 7.14	E2: 58.33
			L3: 42.86	E3: 25
			L4: 0	
			L4+: 21.43	

Abbreviations: CD, Crohn's disease; CON, control/healthy donors; HBI Harvey‐Bradshaw Index; PMS, partial Mayo score; UC, ulcerative colitis.

### 2.2 Flow cytometry of human cells

For the analysis of integrin expression, human PBMCs were incubated with specific antibodies against CD14 (PE, HCD14), CD16 (APC/Cy7, 3G8), β7 integrin (PerCP/Cy5.5, FIB27), αE integrin (PE/Cy7, Ber‐ACT8, all BioLegend), AF488‐labelled β7 integrin (9D8, Genentech) and AF647‐labelled etrolizumab‐s (FIB504, Genentech) for 15 min at 4°C. For labelling, we used the AF488‐labelling kit and the AF647‐labelling kit (ThermoFisher) according to the manufacturer instructions.

Fluorescently labelled cells were fixed with FixPerm (eBioscience) and analyzed by MACSQuant Analyzer 10 (Miltenyi Biotec) and Flow Jo software (v10.8.1).

### 2.3 Fluorescence‐activated cell sorting

For the isolation of classical and non‐classical monocytes from the peripheral human blood, PBMCs were incubated with specific antibodies against CD14 (PE, HCD14), CD16 (APC/Cy7, 3G8), CD56 (FITC, HCD56, all BioLegend), CD3 (VioGreen, REA613) and CD19 (VioBlue, LT19, both Miltenyi) and sorted on Astrios EQ Sorter (Beckman Coulter).

### 2.4 Dynamic adhesion assay

Miniature glass capillaries (Vitrocom) were connected to plastic tubing and coated with 5 µg/ml rhMAdCAM‐1‐Fc‐chimera (R&D Systems) in coating buffer (150 mM NaCl, 10 mM HEPES) and then blocked with 5 % bovine serum albumin (BSA, Pan Biotech).^28^

Fluorescence‐activated cell sorting (FACS)‐sorted CD3^−^CD19^−^CD56^−^CD14^+^CD16^−^ CLMs and CD3^−^CD19^−^CD56^−^CD14^−^CD16^+^ NCMs were labelled with carboxyfluorescein succinimidyl ester (Life Technologies). Then, the cells were incubated with or without 10 µg/ml of etrolizumab‐s in RPMI 1640 supplemented with 10 % fetal calf serum (FCS, PAN Biotech) and 1 % P/S (Penicillin/Streptomycin, Gibco) for 1 h at 37°C. Next, the cells were diluted in adhesion buffer (pH 7.4; 150 mM NaCl, 1 mM HEPES, 1 mM CaCl_2_, 1 mM MgCl_2_, 1 mM MnCl_2_) and perfused through the previously MAdCAM‐1‐coated capillaries for 3 min using a peristaltic pump (Lab V3, Shenchen). The capillaries were rinsed to remove non‐adhering cells and then imaged with a Leica Inverted Microscope (DMi8) with 20x magnification. Adhering cells on eight high power fields were quantified for each capillary.

### 2.5 Isolation and primary culture of mouse bone marrow‐derived monocytes

Bone marrow cell suspensions were isolated as previously described.^27^ Briefly, bone marrow cells were collected by flushing femurs and tibias of 8–10‐week‐old C57Bl/6J mice (Janvier) with a 27 3/4G needle through a 70 µm nylon strainer. The remaining cells were supplemented with 20 µg/l recombinant mouse M‐CSF (Miltenyi) and 6−8 million cells per well were seeded in six well cell culture plates for suspension cells (Sarstedt). The cells were cultured in a humidified incubator at 37°C and 5 % CO_2_ in complete RPMI 1640 supplemented with 10 % FCS and 1 % P/S for 4 days. They were harvested with a cell scraper and repeatedly washed with phosphate buffered saline (PBS) with 0.5 % BSA.

For further flow cytometry analysis, one million cultivated cells were stained for viability using the eBioscience Viability dye eFluor 506 (Invitrogen) and unspecific binding was blocked using Fc Blocking Reagent (Miltenyi). Cell surface marker staining was performed as described above by incubation with the following antibodies: Ly6C (APC‐Cy7, HK1.4), Cx3cr1 (PacBlue, SA011F11), Cd103 (PE/Cy7, 2E7), Cd115 (BV605, AF598), Cd49d (FITC, R1‐2), and β7 integrin (APC, FIB504, all BioLegend). Samples were fixed with 300 µl BD cell fix (BD Bioscience) for 1 h at 4°C, and analyses were performed on an LSR Fortessa (BD) instrument and with Flow Jo software (v10.8.1).

For the isolation of classical and non‐classical monocytes, the cultivated bone marrow cells were incubated with specific antibodies against Cd115 (BV605, AF598), Ly6C (APC‐Cy7, HK1.4) and Cx3cr1 (Pacific Blue, SA011F11, all BioLegend) and sorted on Astrios EQ Sorter (Beckman Coulter).

### 2.6 Static adhesion assays of human and mouse cells

Eight well immunofluorescence slides (Thermo Scientific) were coated with 5 µg/ml human E‐Cadherin (BioLegend) in coating buffer (150 mM NaCl, 10 mM HEPES) and then blocked with 5% BSA. FACS‐purified CLM and NCM were incubated either with 10 µg/ml etrolizumab‐s (Genentech), with 10 µg/ml vedolizumab (Takeda) or with PBS in RPMI 1640 with 10 % FCS and 1 % P/S for 1 h at 37°C. Subsequently, the cells were diluted in adhesion buffer (pH 7.4; 150 mM NaCl, 1 mM HEPES, 1 mM CaCl_2_, 1 mM MgCl_2_, 1 mM MnCl_2_) and transferred on the slides. After 30 min of incubation at 37°C non‐adhering cells were carefully rinsed away with PBS. Next, adhering cells were fixed with 4 % paraformaldehyde (PFA) and nuclei were stained with Hoechst (Life Technologies). Slides were analyzed by fluorescence microscopy (Leica DM60000B) at 10× magnification and adhering cells were counted with ImageJ (NIH). Additionally, a representative high power field of each well was documented with confocal microscopy (Leica SP8) at 40× magnification.

Mouse static adhesion assays were performed similarly. Briefly, the immunofluorescence slides were coated with 5 µg/ml mouse E‐Cadherin (R&D Systems) and then blocked with 5 % BSA. FACS‐purified Ly6C^high^Cx3cr1^low^ CLMs and Ly6C^low^Cx3cr1^high^ NCMs were incubated with or without 10 µg/ml anti‐β7 integrin (FIB504, Genentech) or anti‐α4β7 antibody (DAT32, BioXCell), respectively.

### 2.7 Mice

C57BL/6J (WT) mice were purchased from Janvier, Cd68^+/GFP^ (C57BL/6‐Tg(CD68‐EGFP)1Drg/J) were purchased from Jackson. Homozygous Ccr2^RFP/RFP^ (B6.129(Cg)‐Ccr2^tm2.1Ifc^/J) mice were received from the Jackson Laboratory and were bred in house to C57BL/6J mice to obtain heterozygous Ccr2^+/RFP^ littermates. Cx3cr1‐Cre x B6.Cg‐Gt(ROSA)26Sor^tm9(CAG‐tdTomato)HzeMGI^ mice (Cx3cr1‐tdTomato) were available in house. All animals were bred and housed in individually ventilated cages with a regular 12‐h day‐night cycle and used for experiments according to approval of the Government of Lower Franconia.

### 2.8 In vivo wound healing model

In vivo wound healing was performed as previously described.^27^ Briefly, mice were anesthetized with isoflurane. Intestinal mucosal wounds were inflicted with a biopsy forceps during colonoscopy, which was performed with a mini‐endoscopy system (Karl Storz). The wound diameters were documented by colonoscopy from day 0 to 7, measured with Image J and related to the initial wound diameter at day 0. Where indicated, mice were treated daily either with anti‐β7 integrin antibody (FIB504, Genentech), anti‐α4β7 integrin antibody (DAT32, BioXCell) or IgG isotype control antibody (Sigma) (100 µg/µl in PBS, i.p.).

### 2.9 Immunohistochemistry

Tissue for immunohistochemistry was obtained at day 5 of wound healing experiments. A biopsy punch was used to obtain wound areas, which were frozen and cut in OCT compound (Tissue Tek, Sakura).

Cryosections were fixed with 4 % PFA. Unspecific binding sites were blocked with Avidin/Biotin‐Blocking‐Kit (Vector Laboratories) and with protein blocking reagent (ROTI®ImmunoBlock, Roth) supplemented with 5 % BSA (PAN‐Biotech) and 20 % goat serum (Vector Laboratories). The slides were subsequently permeabilized with 0.1 % triton X and were incubated with primary antibodies against F4/80 (BM8, BioLegend), Cd68 (Polyclonal, abcam), iNos (Polyclonal, abcam), Cd163 (TNKUPJ, Thermofisher), Cd206 (polyclonal, abcam) and Arginase‐1 (polyclonal, Novus Biologicals). Goat ant‐rabbit AF488 (Invitrogen), goat anti‐rat biotin followed by streptavidin‐Cy3 (both BioLegend), goat anti rat AF488 (abcam) and goat anti‐rabbit Cy3 (Millipore) were used for detection. Nuclei were stained with Hoechst (Life Technologies). Analyses were performed with fluorescence microscopy (Leica DM6000B) and with confocal microscopy (Leica SP8).

### 2.10 In vivo model of homing to the inflamed gut

Colitis was induced in C57Bl/6J mice by supplementing their drinking water with 1 %–1.5 % dextran sulfate sodium (DSS, MP Biomedicals) for 7 days followed by 3 days of normal water.

Bone marrow cells were isolated from C57Bl/6J donor mice and cultured as mentioned above. After harvesting, NCMs were sorted and stained with CellTrace FarRed (Invitrogen) at 37°C for 20 min. One to 2.7 million labelled cells were injected into the ileocolic artery of mice with DSS colitis together with 100 µg anti‐β7 antibody (FIB504, BioXCell or Genentech), anti‐α4β7 antibody (DATK32, BioXCell) or IgG isotype (Sigma), respectively. After 2 h, lamina propria mononuclear cells (LPMCs) were isolated from the colon using the Lamina Propria Dissociation Kit (Miltenyi Biotec) according to the manufacturer's instructions followed by Percoll density gradient centrifugation (GE Healthcare). The LPMCs were stained for viable cells and/or Cd11b^+^ cells (Pacific Blue, M1/70, BioLegend), fixed with BD cell fix or FixPerm and the FarRed^+^ non‐classical monocytes homed to the gut were quantified by flow cytometry.

### 2.11 Ex vivo multiphoton microscopy of wound tissue

Ccr2^+/RFP^, Cd68^+/GFP^ and Cx3cr1‐tdTomato reporter mice were used to perform ex‐vivo multiphoton microscopy (MPM) to examine the infiltration of different cell types into wounds obtained from the colon at days 1, 3 or 5 after wounding. In total, 73 3D image stacks were recorded and analyzed (day 1, 3 and 5 after wounding). Fresh tissue samples containing wounds and surrounding tissue were dissected, kept in cold PBS, and multiphoton microscopy was performed on the same day. A water immersion objective (HC Fluortar L 16x/.6 W VISIR, Leica microsystems) was used on an upright multiphoton microscope (TriMScope II, LaVision BioTec) at a wavelength of 810 nm. The optical filters were chosen to target second harmonic generation from collagen fibers (ET405/20, Chroma), and natural autofluorescence from NADH in mucosal epithelial cells (450/70 BrightLine HC, Semrock Inc.).^29^, ^30^ In addition, a third filter was used to target specific fluorescence from either GFP (525/50), tdTomato (572/35) or RFP (595/40) expressed by the respective reporter macrophage/monocyte subtype. MPM stacks were recorded at an axial spacing of 2 µm. The lateral image size was 682 × 682 µm^2^, and the pixel size was 1.33 µm. Image contrast was adjusted manually using Fiji/ImageJ upon visual inspection. No further image processing has been used.

### 2.12 RNA‐sequencing analysis

The gene expression profile data GSE152316^20^ were retrieved from the NCBI Gene Expression Omnibus database. The dataset contained RNA isolated from intestinal biopsies of patients with CD in a phase 3 randomized double‐blind placebo‐controlled 14‐week trial of etrolizumab. Only samples that were taken from a region with endoscopically active disease in the colon were included in the downstream analysis. The RNA‐seq read counts per gene of patients before treatment versus the same patients 14 weeks after treatment with etrolizumab or placebo were compared by differential expression analysis. The differential expression analysis was conducted with DESeq2 v1.34.0 in R with default settings. To show differentially expressed genes associated with M2‐like macrophages, a heat map was generated with RStudio using the packages ‘gplots’, ‘RColorBrewer’ and ‘scales’. In addition, a volcano plot was generated with GraphPad Prism (v9.0.2). For further downstream analysis, these significantly regulated genes were used for pathway analysis that was done with the Ingenuity Pathway Analysis software (Qiagen).

### 2.13 Statistics

All statistical analyses were performed with the GraphPad Prism software (v9.0.2). In order to choose the appropriate statistical (parametric or non‐parametric) tests, all data sets were tested for normal distribution with the Shapiro‐Wilk test. In case two groups were analyzed, a paired or unpaired *t*‐test was used for normally distributed data and the Wilcoxon Test was used for not normally distributed dependent data. If the data was dependent, normally distributed and more than two groups were analyzed, a RM one‐way‐analysis of variance with the Geisser‐Greenhouse correction was chosen with the Tukey post hoc test. If the data was not normally distributed, but the samples were dependent, the Friedman Test with the Dunn's post hoc test was performed. For analysis of more than two groups, which were independent and not normally distributed, the Kruskal‐Wallis test with the Dunn's post hoc test was chosen. Outliers were identified with the ROUT test with a Q = 1 %. Error bars display the standard error of the mean in all graphs. If not otherwise indicated, *p* values are stated as follows: *p < .05; **p < .01; ***p < .001, ****p < .0001. To identify the correlation between differentially regulated genes, we performed Spearman correlation with GraphPad Prism software (v9.0.2) and generated heatmaps showing the correlation coefficient r.

## 3 RESULTS

### 3.1 Non‐classical monocytes are a target of etrolizumab‐s

**FIGURE 1:**
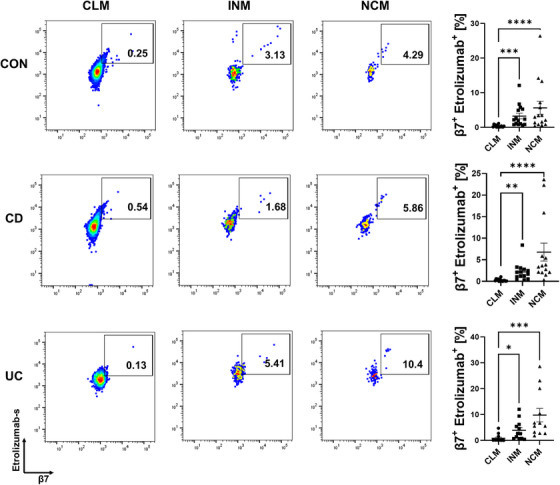
Non‐classical monocytes are a target of etrolizumab‐s. Flow cytometry showing the binding of FIB504 (etrolizumab‐s) to classical, intermediate and non‐classical monocytes in control donors (*n* = 15), patients with Crohn's disease (CD) (*n* = 14) and ulcerative colitis (UC) (*n* = 12). Representative (left panels) and quantitative (right panels) flow cytometry. CLM, classical monocytes; INM, intermediate monocytes; NCM, non‐classical monocytes; *p < 0.05; **p < 0.01; ***p < 0.001; ****p < 0.0001.

We had previously shown that non‐classical human monocytes express α4β7 integrin.^27^ To investigate whether they also express αEβ7, we performed flow cytometry on PBMCs. Monocyte subsets were determined based on the expression of CD14 and CD16 (Figure S1A). Similar to α4β7, co‐expression of αE and β7 integrin was clearly increased on CD14^+^CD16^++^ non‐classical monocytes (NCM) and CD14^++^CD16^+^ intermediate monocytes (INM) compared to CD14^++^CD16^−^ classical monocytes (CLM; Figure S1B,C). Consistently, when we stained cells with etrolizumab‐s (differing from etrolizumab in the backbone, but sharing the same antigen recognition site), etrolizumab‐s bound to a substantially larger fraction of non‐classical and intermediate monocytes compared to classical monocytes (Figure 1, Figure S1D). Together, these data suggested that αEβ7 is expressed on non‐classical monocytes and a target of etrolizumab.

### 3.2 Etrolizumab‐s blocks the interaction of non‐classical monocytes with MAdCAM‐1 and E‐Cadherin

**FIGURE 2:**
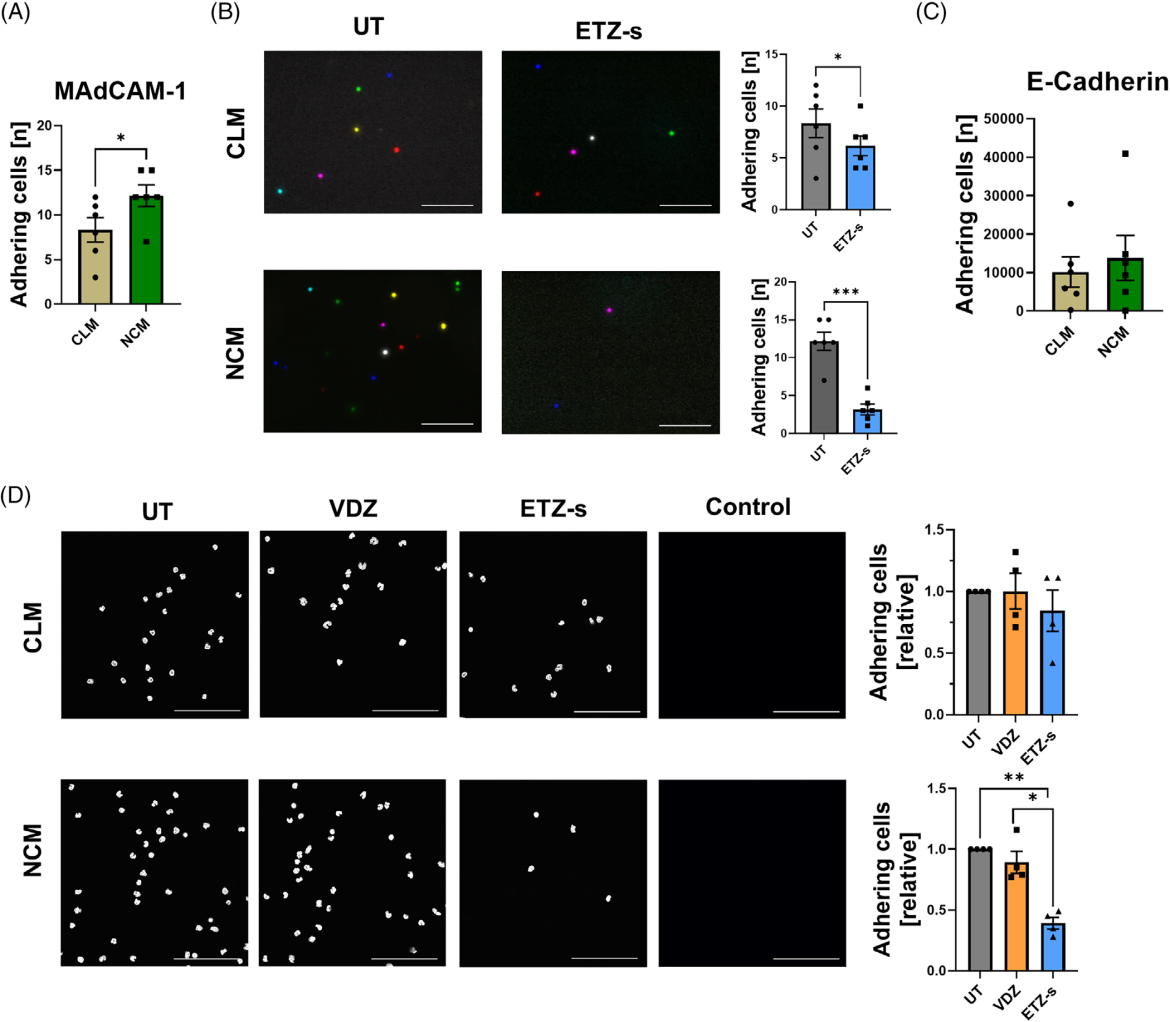
Monocyte‐expressed β7 integrin is functional for interaction with MAdCAM‐1 and E‐Cadherin. (A) Quantitative analysis of the dynamic adhesion of human CD14^++^CD16^−^ classical (CLM) and CD14^+^CD16^++^ non‐classical monocytes (NCM) to mucosal vascular addressin cell adhesion molecule 1 (MAdCAM‐1) (*n* = 6). (B) Dynamic adhesion of CLM and NCM treated without (UT) or with etrolizumab‐s (ETZ‐s) and perfused through capillaries coated with MAdCAM‐1. Left panels: representative merged images from eight high‐power fields, scale bar 100 µm. Right panels: quantification (*n* = 6). (C) Quantification of static adhesion of NCM compared to CLM to E‐Cadherin (*n* = 6). (D) Static adhesion of CLM and NCM treated without (UT) or with ETZ‐s or with Vedolizumab (VDZ) to wells coated with E‐cadherin; scale bar 100 µm. Left panels: representative images from E‐cadherin coated wells. Right panels: quantification (*n* = 4). *p < 0.05; **p < 0.01; ***p < 0.001.

Next, we aimed to explore the function of αEβ7 expressed on human non‐classical monocytes and the potential of etrolizumab‐s to block β7‐dependent functions. Accordingly, we FACS‐sorted human non‐classical and classical monocytes from the peripheral blood, labelled them and used them in dynamic adhesion assays. Upon perfusion through capillaries coated with the α4β7 integrin ligand MAdCAM‐1, more non‐classical than classical monocytes adhered (Figure 2A). Treatment with etrolizumab‐s in vitro clearly reduced the adhesion of non‐classical monocytes but had only marginal effects on classical monocytes (Figure 2B). Thus, etrolizumab‐s blocked α4β7‐dependent adhesion of non‐classical monocytes to MAdCAM‐1.

To interrogate the interaction of αEβ7 with E‐Cadherin, we used static adhesion assays, in which we coated slides with E‐Cadherin and determined binding of non‐classical and classical monocytes. By trend, more non‐classical than classical monocytes bound to E‐Cadherin (Figure 2C). Moreover, etrolizumab‐s but not the anti‐α4β7 integrin antibody vedolizumab selectively blocked the adhesion of non‐classical, but not classical monocytes to E‐Cadherin (Figure 2D).

Together, these data showed that αEβ7 integrin on non‐classical monocytes is functional for the interaction with E‐Cadherin and etrolizumab‐s functionally targets both α4β7 and αEβ7 on non‐classical monocytes.

### 3.3 Increased reduction of non‐classical monocyte accumulation in the inflamed gut in vivo with anti‐β7 compared to anti‐α4β7 treatment

**FIGURE 3:**
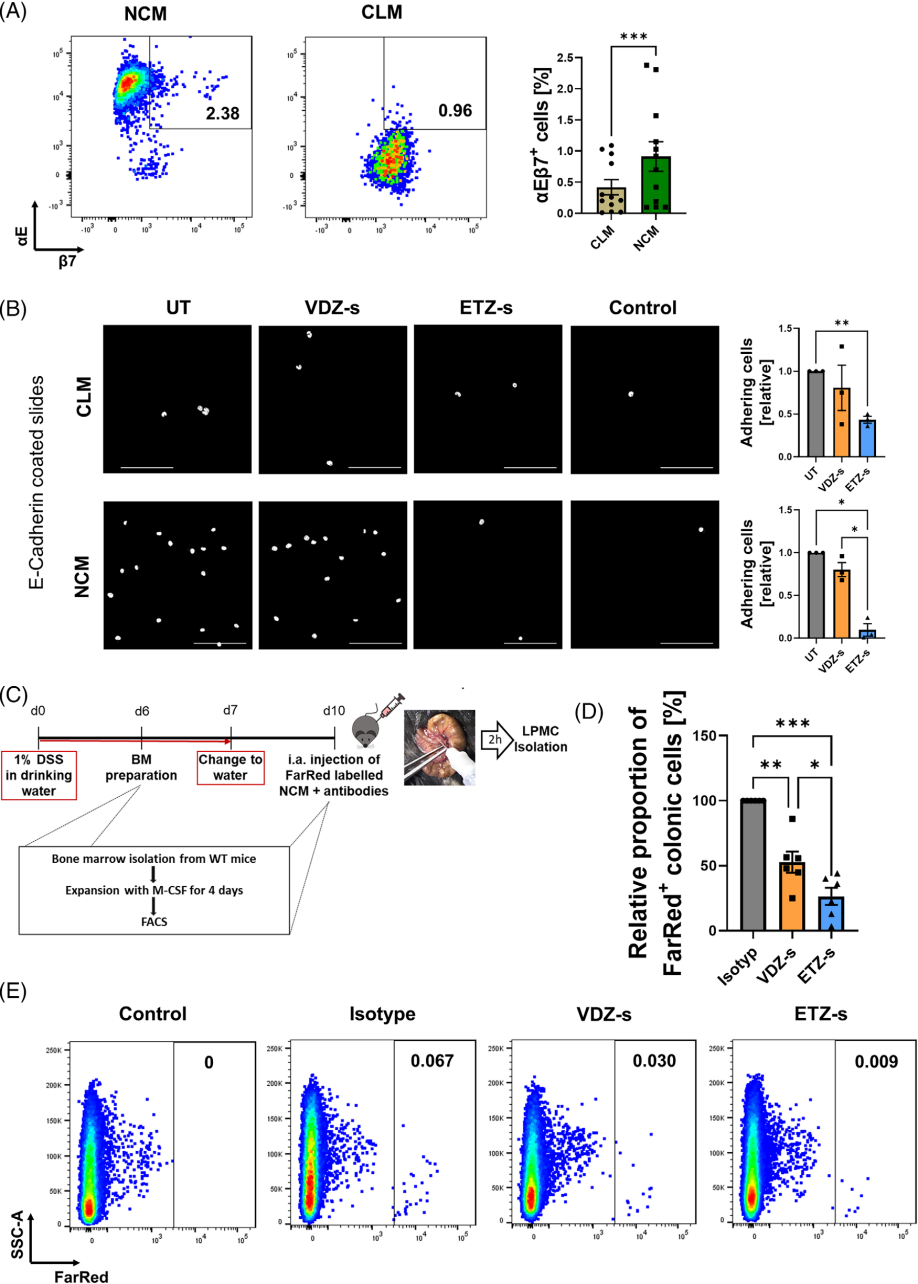
More efficient blockade of non‐classical monocyte recruitment to the gut in vivo by etrolizumab‐s compared to vedolizumab‐s. (A) Analysis of αEβ7 expression on mouse Ly6C^high^Cx3cr1^low^ classical (CLM) and Ly6C^low^Cx3cr1^high^ non‐classical monocytes (NCM). Representative (left panels) and quantitative (right panels) flow cytometry (*n* = 12). (B) Static adhesion of mouse CLM and NCM treated without (UT) or with anti‐β7 (ETZ‐s) or with anti‐α4β7 (VDZ‐s) to E‐Cadherin. Left panels: representative images; scale bar: 100 µm. Right panels: quantification (*n* = 3). (C–E) In vivo homing of NCM after adoptive transfer to the ileocolic artery in combination with anti‐β7 antibody, anti‐α4β7 antibody or isotype control treatment. (C) Schematic depiction of the experimental setup. (D) Quantitative flow cytometry of fluorescently labelled NCM homing to the gut normalized to the isotype control (*n* = 6). (E) Representative flow cytometry of Cd11b^+^FarRed^+^ cells in the colon 2 h after i.a. transfer. Outliers were identified using the ROU test. *p < 0.05; **p < 0.01; ***p < 0.001.

Since we required experimental mouse models to further investigate the role of etrolizumab‐s for monocyte accumulation in the gut in vivo, we cultured bone marrow monocytes and sorted them into Ly6C^high^Cx3cr1^low^ classical and Ly6C^low^Cx3cr1^high^ non‐classical monocytes (Figure S2). αEβ7 expression on non‐classical monocytes was doubled compared to classical monocytes (Figure 3A). Consistently, in static adhesion assays with recombinant mouse E‐Cadherin, etrolizumab‐s, but not a vedolizumab surrogate (vedolizumab‐s) anti‐α4β7 antibody reduced the adhesion of monocytes. This was clearly more pronounced for non‐classical monocytes (Figure 3B).

We therefore wondered whether the additional blockade of αEβ7 by anti‐β7 antibodies might be more effective than anti‐α4β7 to reduce the accumulation of non‐classical monocytes in vivo. To this end, we transferred labelled non‐classical monocytes to mice with DSS colitis and analyzed the accumulation in the inflamed gut upon treatment with anti‐β7 or anti‐α4β7 (Figure 3C). In consistence with previous observations, anti‐α4β7 treatment significantly reduced non‐classical monocyte infiltration. However, anti‐β7 treatment led to an even greater reduction of non‐classical monocyte accumulation (Figure 3D,E).

### 3.4 Anti‐β7 treatment delays experimental wound healing more than anti‐α4β7 treatment

Since it has been previously shown that non‐classical monocytes preferentially differentiate into macrophages with a wound healing phenotype in the gut^27^ as well as in other organs,^31^, ^32^ we now aimed to find out, which implications these effects on monocyte trafficking have for intestinal wound healing.

**FIGURE 4:**
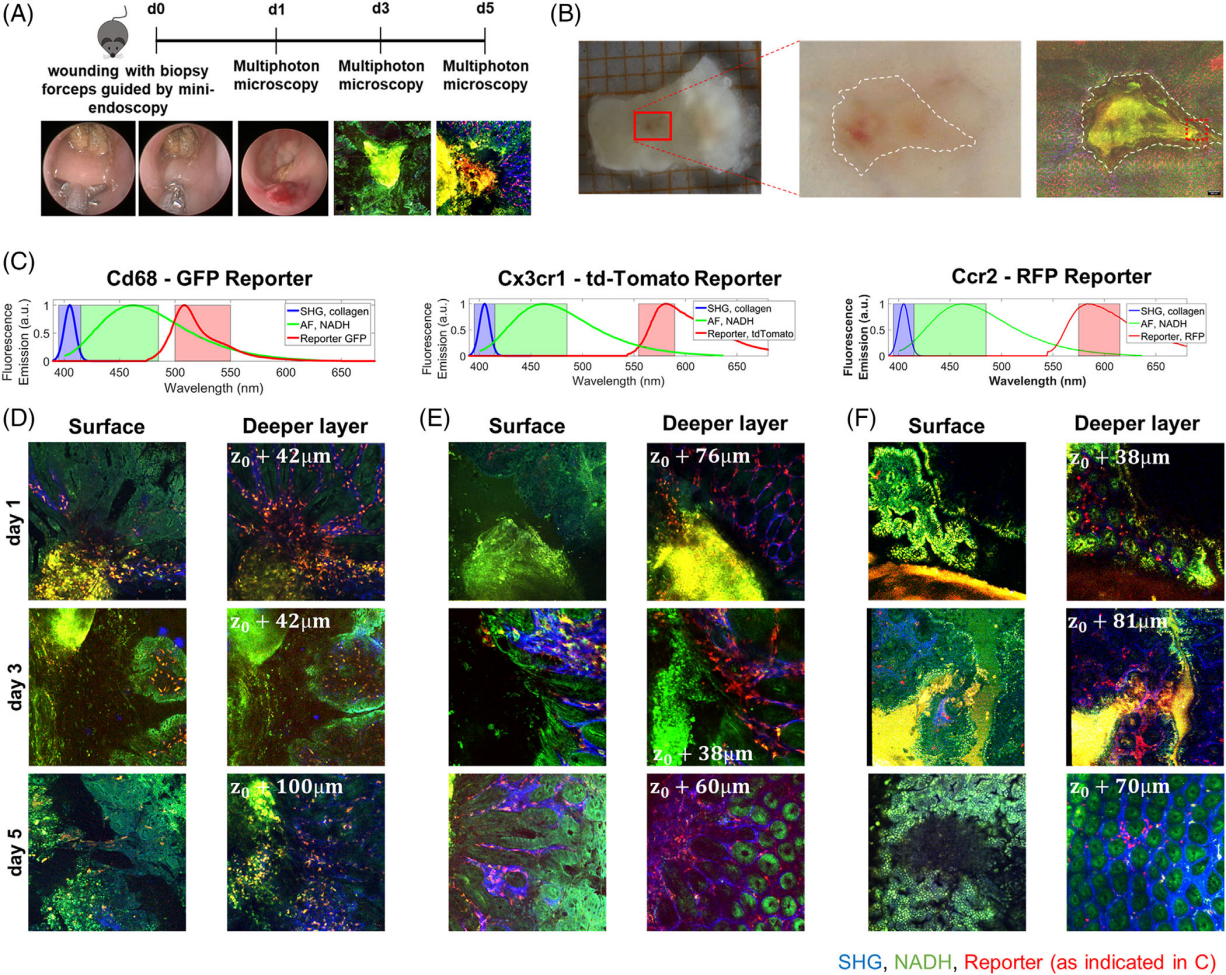
Deep tissue 3D Multiphoton imaging of tissue samples from Ccr2^+/RFP^, Cd68^+/GFP^ and Cx3cr1‐tdTomato reporter mice. (A) Schematic depiction of the experimental procedure. Fresh colon samples were investigated 1, 3 or 5 days after inflicting mucosal wounds with a biopsy forceps guided by mini‐endoscopy. (B) White light microscopy shows reddish blood clots on day 1, but without molecular contrast. Multiphoton microscopy visualizes second harmonic generation (SHG) from collagen fibers (blue), the natural autofluorescence from NADH in epithelial cells (green) and the specific fluorescence from either GFP, tdTomato or RFP reporter macrophages/monocytes (red). From the larger wound bed, several locations at the rim (red box) were selected for highly resolved 3D imaging. (C–F) Deep tissue 3D multiphoton images of mucosal wounds in reporter mice at different time points. (C) Spectral channel of collagen (SHG), NADH (AF) and the respective reporters for Cd68 (GFP), Cx3cr1 (tdTomato) and Ccr2 (RFP). (D–F) Representative images from wounded tissue on different days of Cd68‐GFP (D), Cx3cr1‐tdTomato (E) and Ccr2‐RFP (F). The right column shows a superficial image plane and the left column a deeper tissue plane of the same 3D image volume. The wound pit is always shown at the left side of the image. On day 1 after wounding, GFP^+^ cells start to infiltrate the wound bed, while fewer tdTomato^+^ and RFP^+^ cells are present. tdTomato^+^ cells start to infiltrate into the wound area on day 3 and further increase on day 5, while only few RFP^+^ cells are present at this time point.

First, we explored the temporospatial regulation of monocyte subset infiltration into wound beds. To this end, we used reporter mice for the pan‐macrophage marker Cd68, the classical monocyte marker Ccr2 and the non‐classical monocyte marker Cx3cr1. A biopsy forceps was used to create wounds in the colon mucosa and sequential ex vivo multi‐photon microscopy was performed after 1, 3 and 5 days (Figure 4A,B). In addition to the different fluorescent reporters, we used autofluorescence and second harmonics to detect NADH and collagen, respectively (Figure 4C). Cd68^+^ cells were present in the wound area from day 1 on (Figure 4D). However, only few Cx3cr1^+^ cells were present on day 1, and substantial infiltration to the wound area was noted on day 3 and 5 (Figure 4E). To the contrary, infiltration of Ccr2^+^ cells was particularly observed on day 1 and decreased on days 3 and 5 (Figure 4F).

Together, in accordance with the literature, these data suggested that classical monocytes are recruited to intestinal wounds early during wound healing, while non‐classical monocytes giving rise to wound healing macrophages emerge later in the wound beds.

**FIGURE 5:**
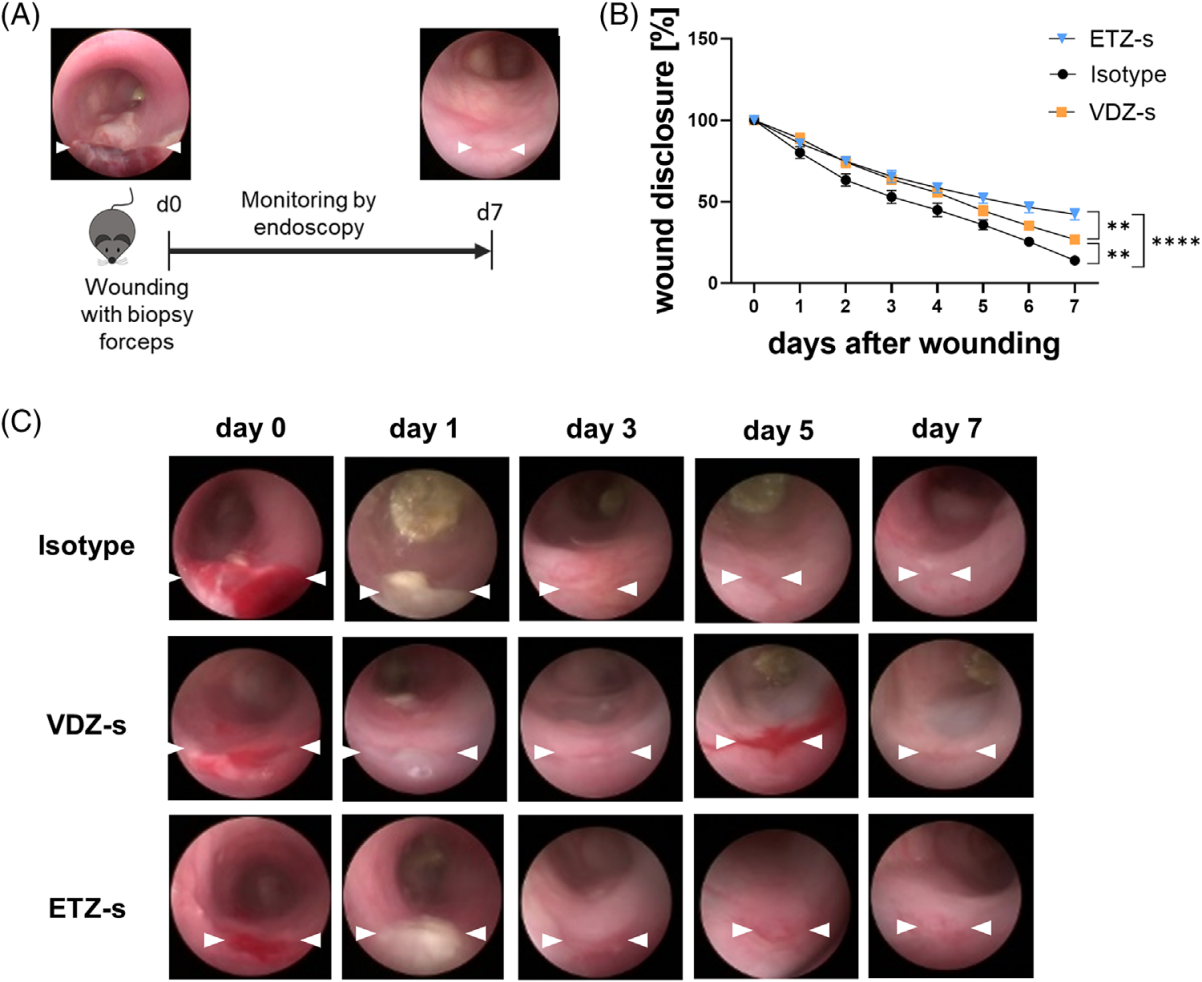
Delayed in vivo wound healing upon treatment with anti‐β7 integrin antibody. (A) Schematic depiction of experimental set‐up. On day 0, wounds were inflicted to the colon of C57BL/6J mice. From day 0 through 7, wound healing was monitored by endoscopy. (B) Quantitative analysis of relative wound diameters over time in mice treated either with anti‐β7 integrin antibody (*n* = 11), anti‐α4β7 integrin antibody (*n* = 8) or with isotype control (*n* = 10). (C) Representative endoscopic images at the indicated time points. **p < 0.01; ****p < 0.0001.

In a next step, we used a well‐established experimental model of in vivo wound healing (Figure 5A) to investigate the effects of etrolizumab‐s and vedolizumab‐s on the restitution of wounds in the colon. As expected based on previous data, vedolizumab‐s slowed down intestinal wound healing. However, etrolizumab‐s even further delayed wound closure (Figure 5B,C).

These findings were consistent with the view that the inhibition of αEβ7‐dependent monocyte retention in the intestine by etrolizumab‐s might be counter‐productive for wound healing.

### 3.5 Anti‐β7 treatment reduces the presence of peri‐lesional macrophages with a wound healing phenotype in the colon

**FIGURE 6:**
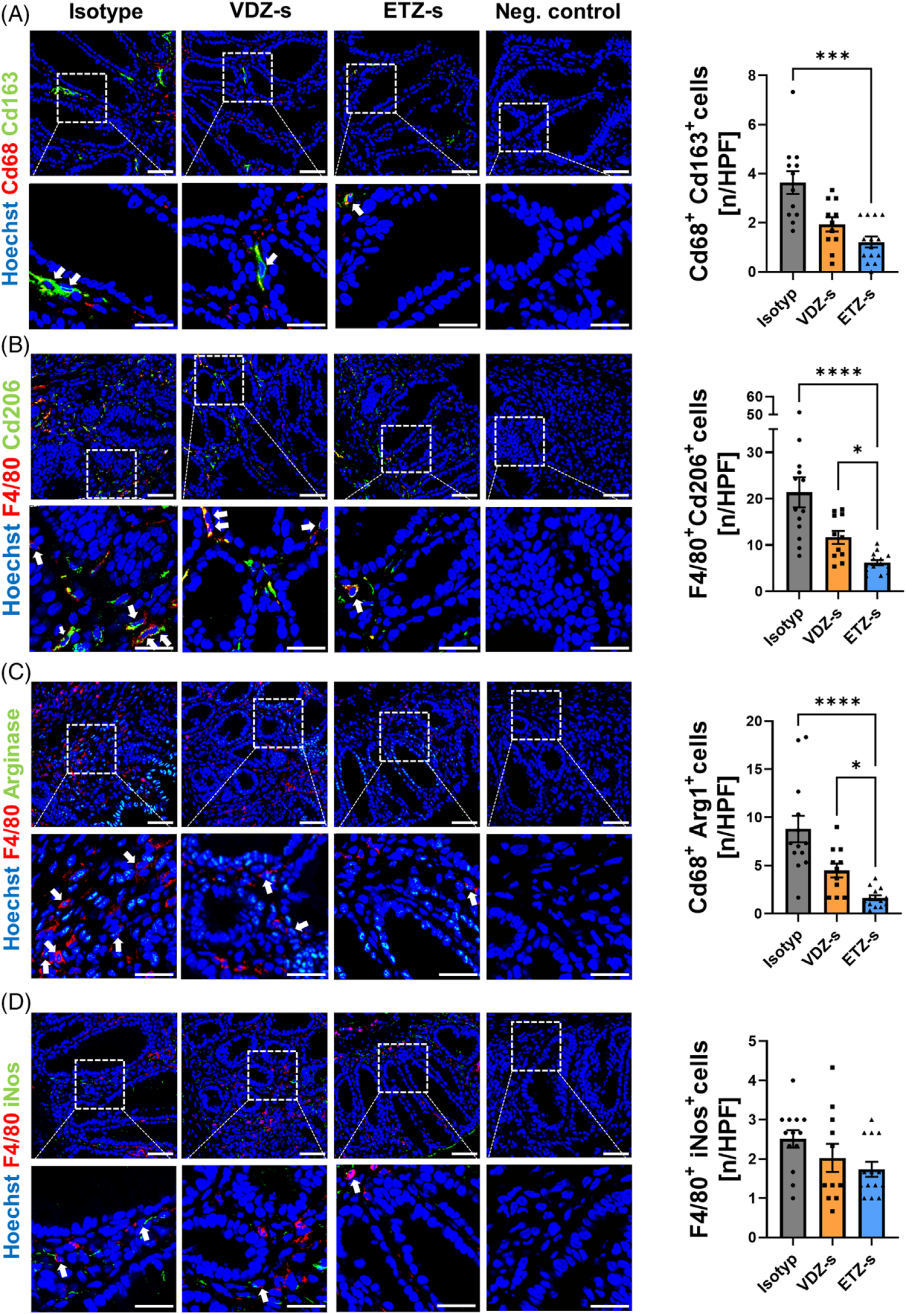
Reduced peri‐lesional presence of wound healing macrophages under treatment with anti‐β7 integrin antibody. Peri‐lesional co‐expression of Cd68 and Cd163 (A), F4/80 and Cd206 (B), F4/80 and Arg1 (C) and F4/80 and inducible nitric oxide synthase (iNos) (D) in intestinal wound areas of C57BL/6J mice treated either with anti‐β7 integrin antibody (*n* = 14), anti‐α4β7 integrin antibody (*n* = 11) or isotype control (*n* = 13). Tissue was collected 5 days after wounding. Representative (left) and quantitative immunohistochemistry (right); scale bars 50 µm (upper panels), 25 µm (lower panels); HPF, high‐power field. Outliers were identified using the ROU test. *p < 0.05; ***p < 0.001; ****p < 0.0001.

Thus, to find out, whether etrolizumab‐s treatment is associated with altered macrophage composition next to intestinal wound areas, we obtained wounds from the above model and performed immunofluorescence stainings. We used Cd163, Cd206 and Arginase 1 (Arg1) as well‐established markers for a wound healing macrophage phenotype together with the pan‐macrophage markers Cd68 or F4/80. Treatment with etrolizumab‐s significantly reduced the abundance of peri‐lesional Cd68^+^Cd163^+^, F4/80^+^Cd206^+^ and F4/80^+^Arg1^+^ cells. Of note, this effect was even more pronounced than that of vedolizumab‐s (Figure 6A–C). Importantly, the abundance of F4/80^+^iNos^+^ cells was comparable between the groups.

Collectively, this supported the notion that, indeed, pan‐β7 blockade further reduces wound healing macrophage accumulation in the context of wound healing compared with sole α4β7 blockade.

### 3.6 Etrolizumab treatment of patients with CD regulates wound healing pathways in the colon

Finally, we aimed to address the question, whether this might also translate to the human situation.

**FIGURE 7:**
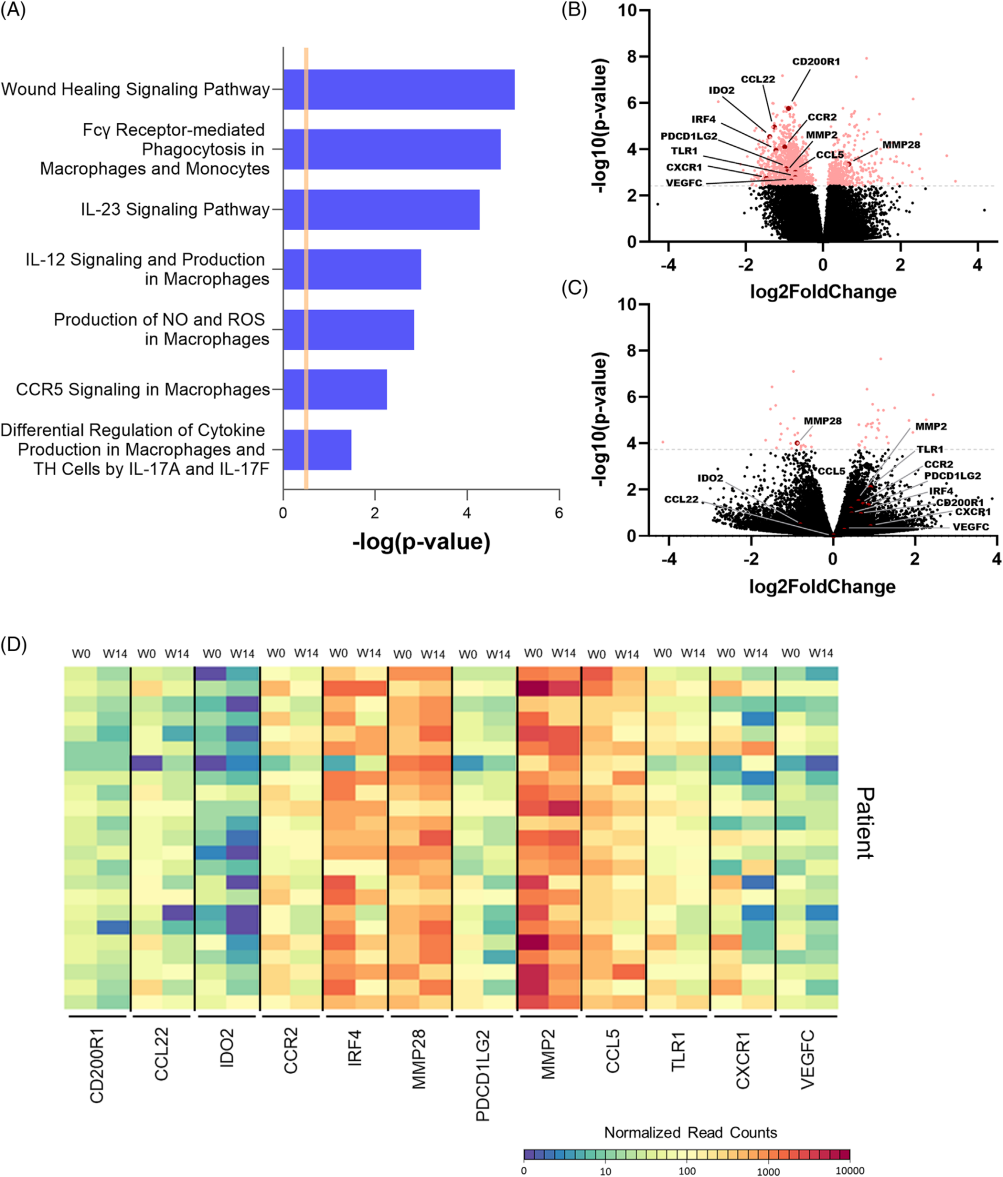
Wound healing macrophage signatures are down‐regulated in the transcriptome of patients with CD under treatment with etrolizumab. (A) Ingenuity pathway analysis of significantly differentially expressed genes (padj. < 0.05) in colon biopsies of patients with CD before and after 14 weeks of etrolizumab treatment. Pathways associated with monocytes and/or macrophages are shown and are predicted to be inhibited. (B) Volcano plot highlighting genes differentially expressed in patients with CD after versus before 14 weeks of etrolizumab treatment. (C) Volcano plot highlight the same genes in patients treated with placebo. The black dots represent genes with no significant difference while the pinkish dots indicate differentially expressed genes based on padj. < 0.05. (D) Heat map depicting selected significantly expressed genes associated with wound healing macrophages as highlighted in (B). Normalized read counts analyzed with DESeq2 are displayed for each patient (*n* = 23).

Accordingly, we mined a publicly available bulk RNA sequencing dataset from patients treated with etrolizumab in a cohort of the BERGAMOT phase III trial of etrolizumab in CD.^20^ We compared the transcriptomics in the colon between baseline and week 14 in patients from the verum group and found 1706 differentially regulated genes. Strikingly, ingenuity pathway analysis of these genes revealed a number of pathways associated with macrophage function being among the top regulated pathways (Figure 7A, Figure S3). Consistently, a huge number of genes related to wound healing macrophage function were down‐regulated in the course of etrolizumab treatment (Figure 7B,D). Importantly, this was not the case in patients from the placebo group (Figure 7C).

Together, these data supported the concept that etrolizumab therapy also interferes with intestinal wound healing in human patients.

## 4 DISCUSSION

The concept of interfering with immune cell trafficking to treat IBD has cued a whole family of therapeutics, of which the anti‐α4β7 integrin antibody vedolizumab and the sphingosine‐1‐phosphate receptor (S1PR) agonist ozanimod have entered clinical routine care.^33^ While further compounds, such as the S1PR agonist etrasimod^34^ or the α4 integrin antagonist AJM300,^35^ have successfully passed phase III trial programs, other candidates failed during clinical development.^17^

One important example is the anti‐β7 integrin antibody etrolizumab. Following very promising results from a phase II trial in UC,^21^ a large phase III trial program was developed with several parallel studies aiming not only at demonstrating efficacy and safety for the treatment of UC and CD, but also at proving superiority over the anti‐TNF antibodies infliximab and adalimumab for the treatment of UC.^18^ However, these trials were concluded with disappointing results and etrolizumab met only few of the primary endpoints.^36^

Given the current knowledge on the mechanism of action of etrolizumab, this seems still surprising: As an anti‐β7 antibody, it has been shown to interfere with the interaction of α4β7 integrin with MAdCAM‐1 as well as of αEβ7 with E‐Cadherin leading to abrogation of T cell homing to as well as T cell retention within the gut.^19^, ^20^, ^37^ In this context, baseline αE expression in the intestine had even be suggested as a biomarker for the prediction of response to etrolizumab.^38^ Yet, apparently, although some biological activity can be concluded from the phase III trials,^22^, ^23^, ^24^, ^25^ these aspects seem either not to be enough for substantial modulation of inflammation in IBD or currently still overlooked aspects limit their pro‐resolving activity.

In this context, the data that we present in this manuscript shed a new light on etrolizumab, since we show that non‐classical monocytes are a target of the antibody. Although an impact of etrolizumab on innate immune cell subsets in general and non‐classical monocytes in special had to be concluded in analogy to what is known for vedolizumab,^27^, ^39^, ^40^, ^41^ these data are the first to show that this effect may go beyond vedolizumab due to the expression of αEβ7 by a fraction of these cells. Indeed, our functional analyses demonstrate that etrolizumab specifically impedes the adhesion of non‐classical monocytes not only to MAdCAM‐1, but also to E‐Cadherin. Importantly, this situation was also conserved in mice allowing us to investigate the consequences for functional processes in the gut in vivo.

Since monocytes recruited to the gut develop into local macrophages^14^ and previous reports consistently suggested a bias of non‐classical monocytes for differentiation into M2‐like wound healing macrophages,^27^, ^31^, ^32^ we chose a model of intestinal wound healing. Indeed, wound healing upon treatment with etrolizumab‐s was further delayed compared with vedolizumab‐s, as was the peri‐lesional presence of macrophages with a wound‐healing phenotype. Consistently, many genes associated with the role of such macrophages in wound healing were down‐regulated during therapy with etrolizumab in a publicly available dataset from the BERGAMOT trial.^20^ It is important to underscore that this link of non‐classical monocytes to M2‐like wound healing macrophages must be understood as a preference and not as exclusive, since also CD14^+^ classical monocytes can give rise to M2‐like macrophages and the conversion of M1 into M2 macrophages is a well‐known phenomenon.^42^ Thus, besides non‐classical monocyte homing, there may also be other sources of wound healing associated macrophages in the gut.

Moreover, it is also essential to acknowledge that cell trafficking and wound healing are multifaceted processes to which various cell types contribute. Thus, it is possible that the effects that we observe are partly due to the crosstalk of other cells with non‐classical monocytes or M2‐like macrophages. For instance, it is known that Ly6C^−^ monocyte‐dervied M2 macrophages contribute to angiogenesis, myofibroblast generation and collagen deposition.^43^ Similarly, M2, but not M1 macrophages are associated with activation of epithelial Wnt signalling and reduced enterocyte differentiation in patients with UC.^44^ Therefore, further studies should address the importance of monocyte and macrophage interaction with other cell types in the context of anti‐β7 treatment.

Another point to consider is that our in vivo data are of correlative nature and do not prove a causal relationship between reduced recruitment of a small subset of non‐classical monocytes and wound healing. In fact, a predominant effect of anti‐β7 treatment on another larger immune cell population might also explain our findings. However, it has previously been shown that αEβ7 expression is largely confined to T cells^45^ and since we have previously shown that our in vivo wound healing model is largely independent of T cells,^27^ there is no such large candidate immune cell population to explain the difference in wound healing between anti‐α4β7‐ and anti‐β7‐treated mice. Importantly, it has previously been shown that small monocyte populations such as S1pr3‐expressing non‐classical monocytes can have a key impact on skin wound healing.^31^ Thus, although we are aware that this is not formally shown, the concept that we propose is well conceivable.

**FIGURE 8:**
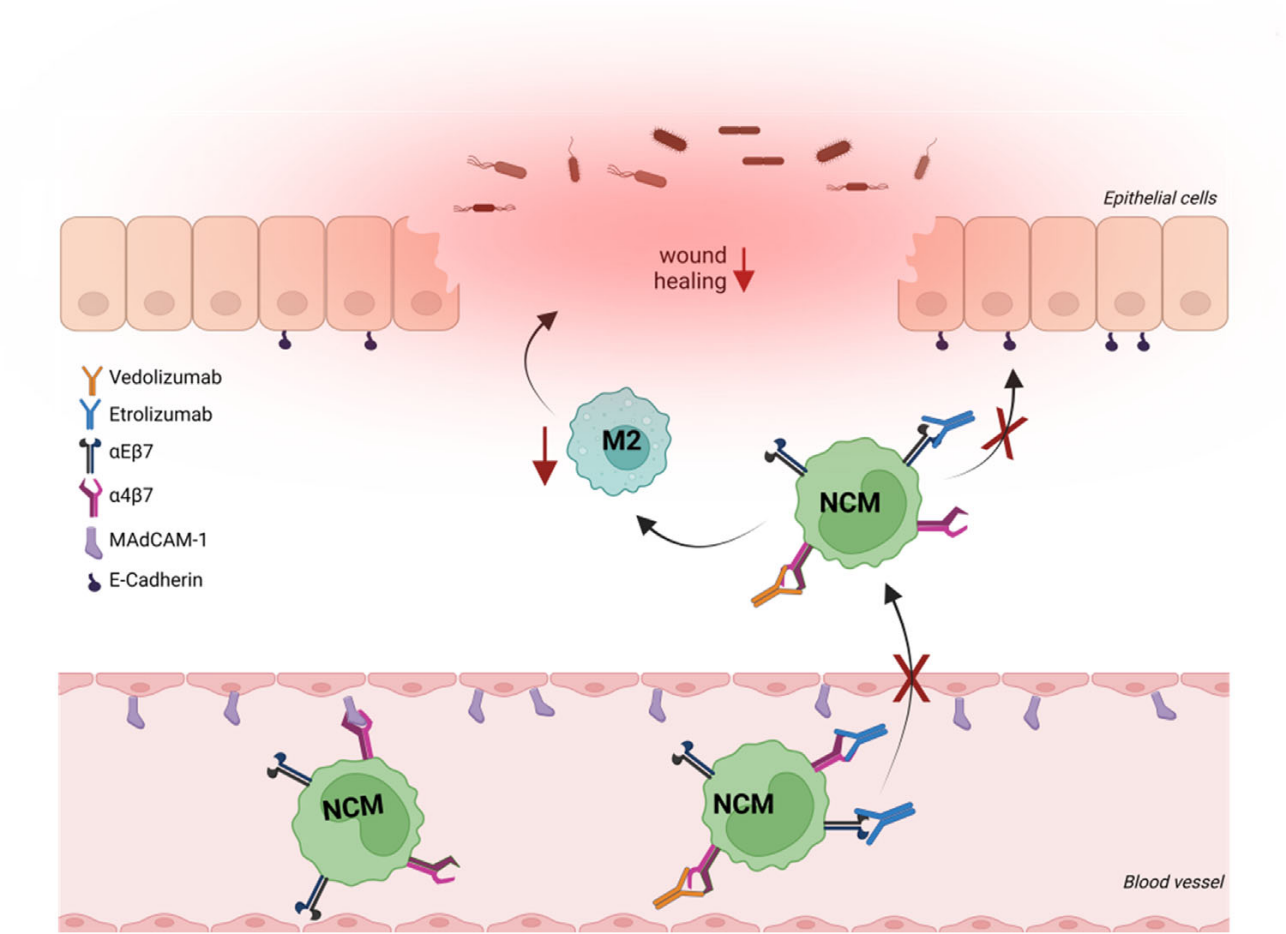
Putative model of the interaction of etrolizumab with non‐classical monocyte recruitment (for details cf. main text). NCM, non‐classical monocytes; figure drawn with licensed BioRender software and modified using Inkscape.

Hence, altogether, our data indicate that the inhibition of non‐classical monocyte recruitment to the gut by etrolizumab may have a negative impact on the restitution of mucosal defects and thus counteract mucosal healing (Figure 8), which might provide a partial explanation for the observations in the phase III trial program.

However, as mentioned above, we had also reported a similar mechanism for vedolizumab,^27^ which is a well‐established and successful pillar of IBD therapy. This leads to the question, why this mechanism should be relevant for etrolizumab, while not relevantly limiting the efficacy of vedolizumab. One obvious answer is that the effects of anti‐β7 treatment on intestinal wound healing were more pronounced than those of anti‐α4β7 therapy due to the broader mechanism of action also covering αEβ7 integrin. However, this is probably not the full explanation, since multi‐faceted interactions between the various aspects of the mechanisms of anti‐trafficking agents need to be considered. For example, we had previously shown for vedolizumab that a preferential blockade of effector T cell recruitment and preserved regulatory T cell gut homing in an ideal exposure window contribute to its efficacy,^28^ while this is not the case for etrolizumab‐s.^46^ Thus, these—and probably further, so far unrecognized aspects—may concertedly lead to the overall limited efficacy of etrolizumab.

A limitation for our study was that we did not have samples from patients treated with etrolizumab at our disposal to directly investigate its effects at tissue‐level. However, this was not possible due to the availability of etrolizumab only in the frame of blinded studies and will probably not be possible, since it is currently very unlikely that etrolizumab will enter the market. Thus, we relied on functional in vitro and in vivo experiments as well as in silico analyses. Although it must be acknowledged that experimental mouse models not fully reflect the human situation and that gene expression trajectories under etrolizumab therapy only provide correlative, but not causal evidence, these are strong tools leading to a completely new vantage point on etrolizumab in the frame of our study.

From a broader perspective, these insights further underscore the complexity of cell trafficking pathways^17^ and the need to consider the interplay of multiple cellular players, when envisioning the functional consequences of targeting a specific molecule involved in immune cell trafficking. Ideally, this should already be addressed in pre‐clinical research and will also require the consideration of optimized experimental models for studying immune cell trafficking.

## 5 CONCLUSIONS

Collectively, our data show for the first time that αEβ7 is expressed and functionally relevant on non‐classical monocytes, making them a likely target for the anti‐β7 antibody etrolizumab. Mechanistically, this seem to lead to delayed macrophage‐dependent wound healing in the gut, which might be one part of the puzzle to explain disappointing observations in recent phase III clinical trials on etrolizumab.

## CONFLICT OF INTEREST STATEMENT

MFN. has served as an advisor for Pentax, Giuliani, MSD, Abbvie, Janssen, Takeda and Boehringer. SZ received speaker's fees from Takeda, Roche, Galapagos, Ferring, Falk, Lilly and Janssen. MFN and SZ received research support from Takeda, Shire (a part of Takeda) and Roche. The other authors declare no conflict of interest.

## Supporting information

Supporting Information

Supporting Information

Supporting Information

Supporting Information
