# Supplementary figures and images for: Anti‐β7 integrin treatment impedes the recruitment on non‐classical monocytes to the gut and delays macrophage‐mediated intestinal wound healing
Source: Clin Transl Med. 2023 Apr 8;13(4):e1233. doi: 10.1002/ctm2.1233 (PMC10082567; doi:10.1002/ctm2.1233)

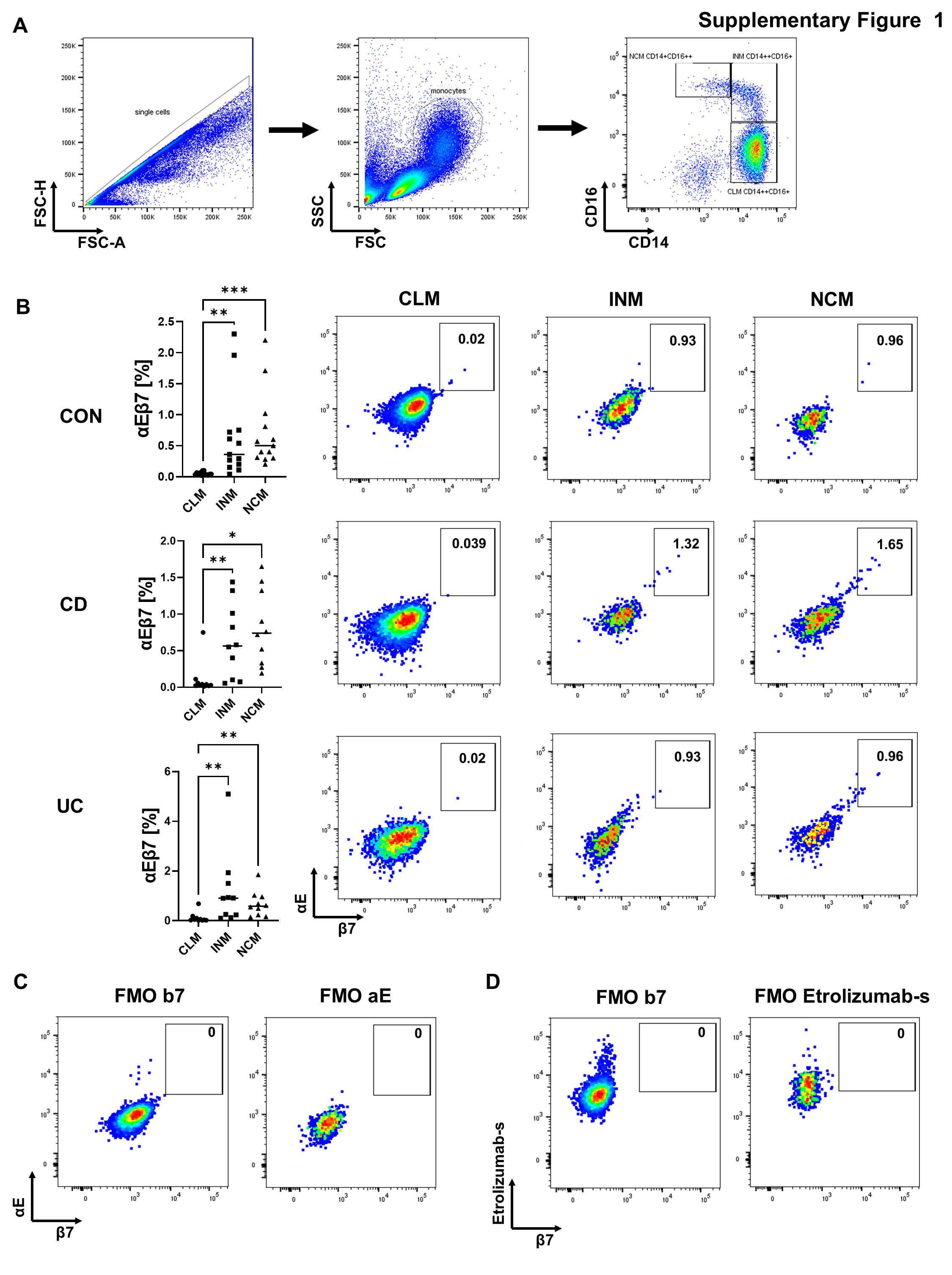

Supplement: Supplementary file 1 — Supporting Information [file CTM2-13-e1233-s003.tif]

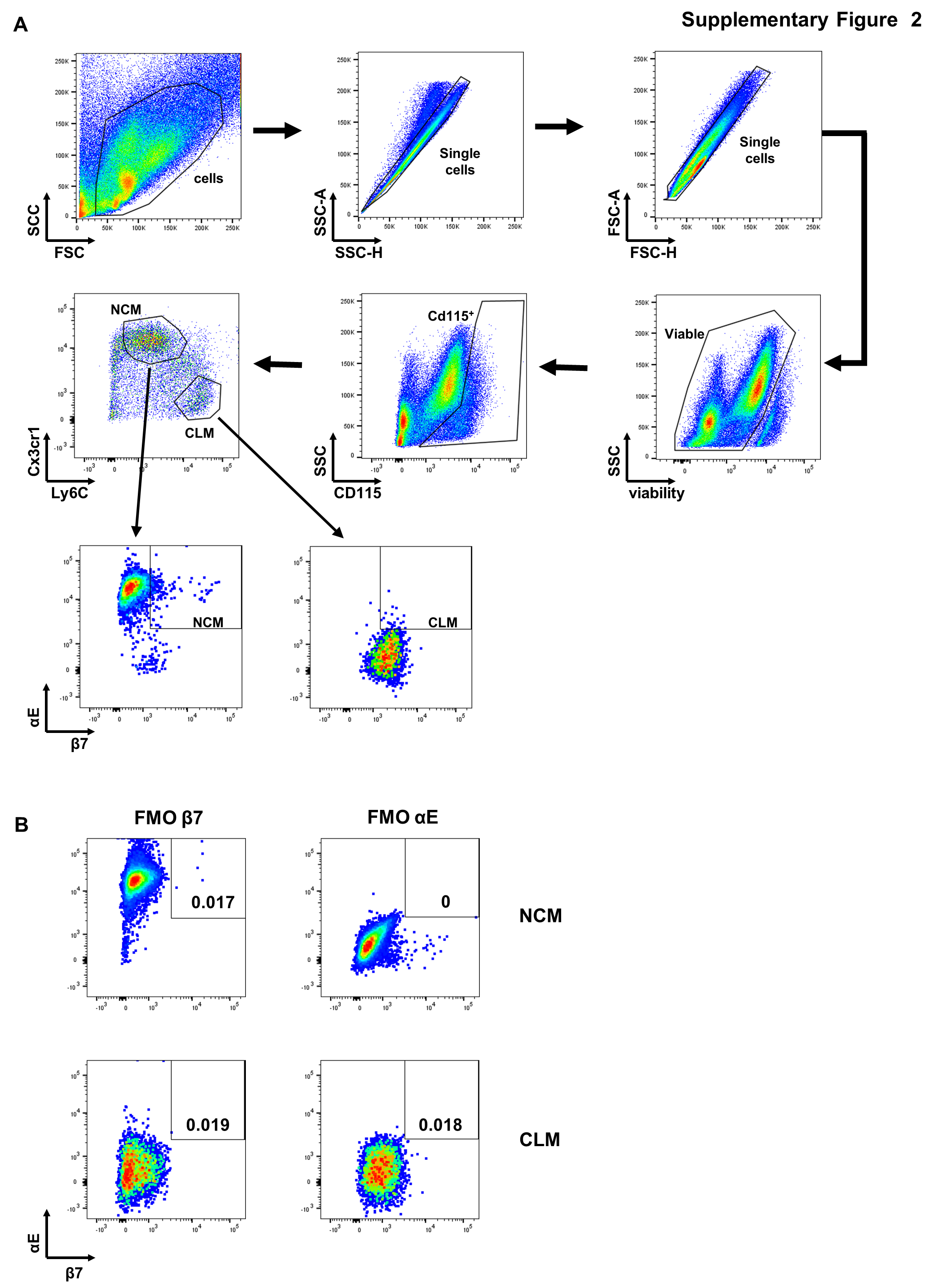

Supplement: Supplementary file 2 — Supporting Information [file CTM2-13-e1233-s004.tif]

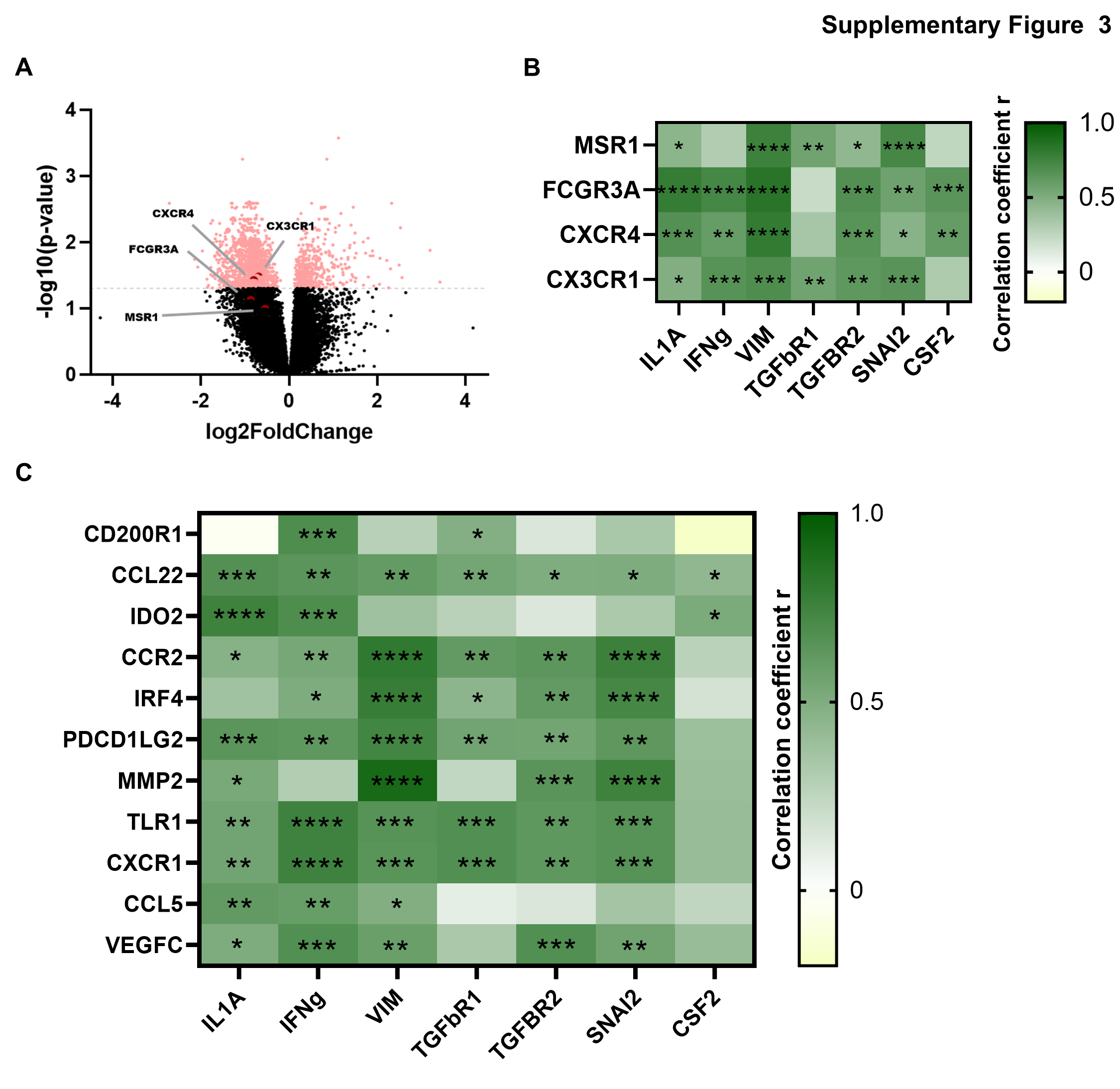

Supplement: Supplementary file 3 — Supporting Information [file CTM2-13-e1233-s001.tif]

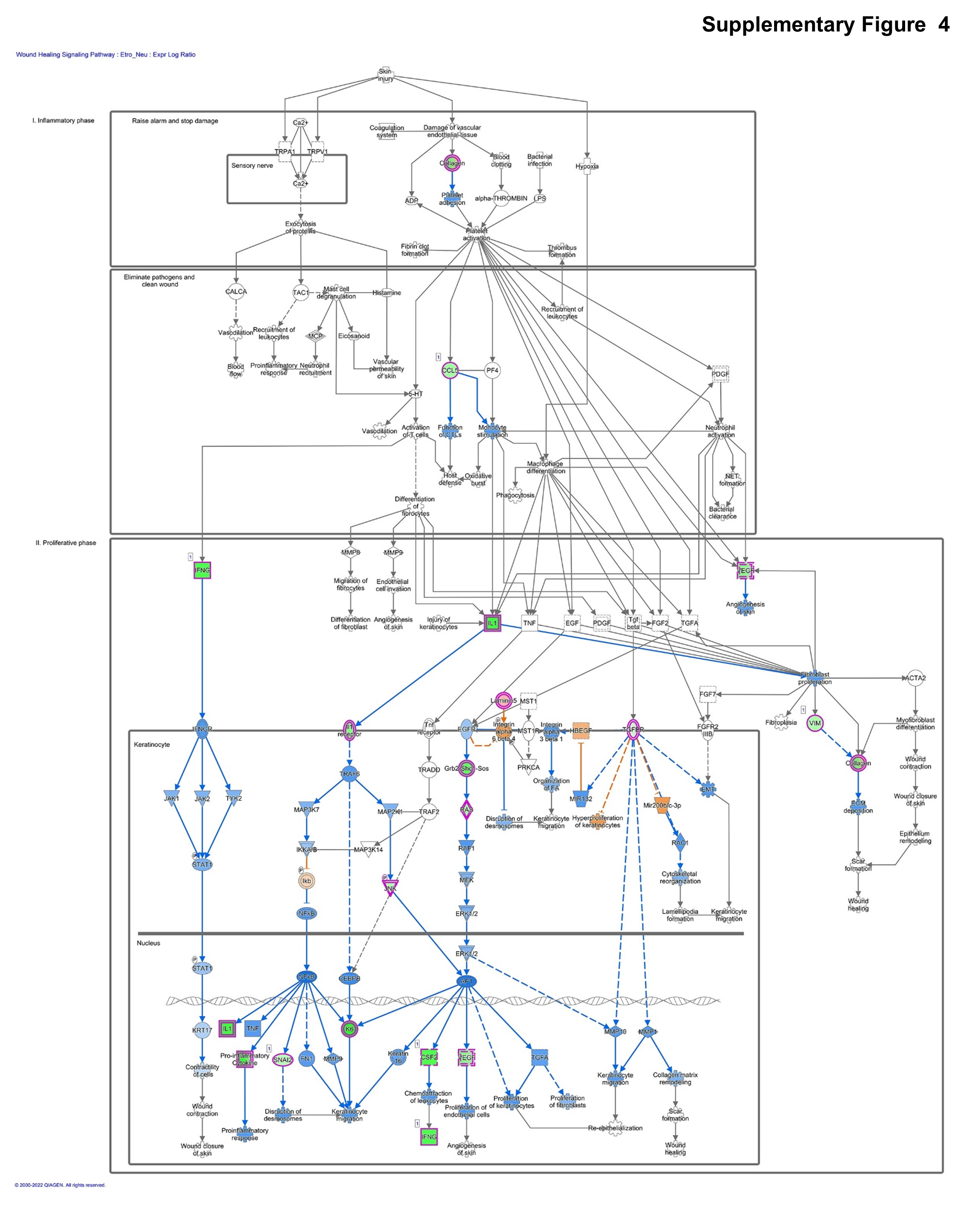

Supplement: Supplementary file 4 — Supporting Information [file CTM2-13-e1233-s002.tif]
